# Supplementary material for: n-Butylidenephthalide recovered calcium homeostasis to ameliorate neurodegeneration of motor neurons derived from amyotrophic lateral sclerosis iPSCs
Source: PLoS One. 2024 Nov 7;19(11):e0311573. doi: 10.1371/journal.pone.0311573 (PMC11542850; doi:10.1371/journal.pone.0311573)
Supplement: S2 Fig — (PDF) [file pone.0311573.s002.pdf]

Fig 1E. G85R

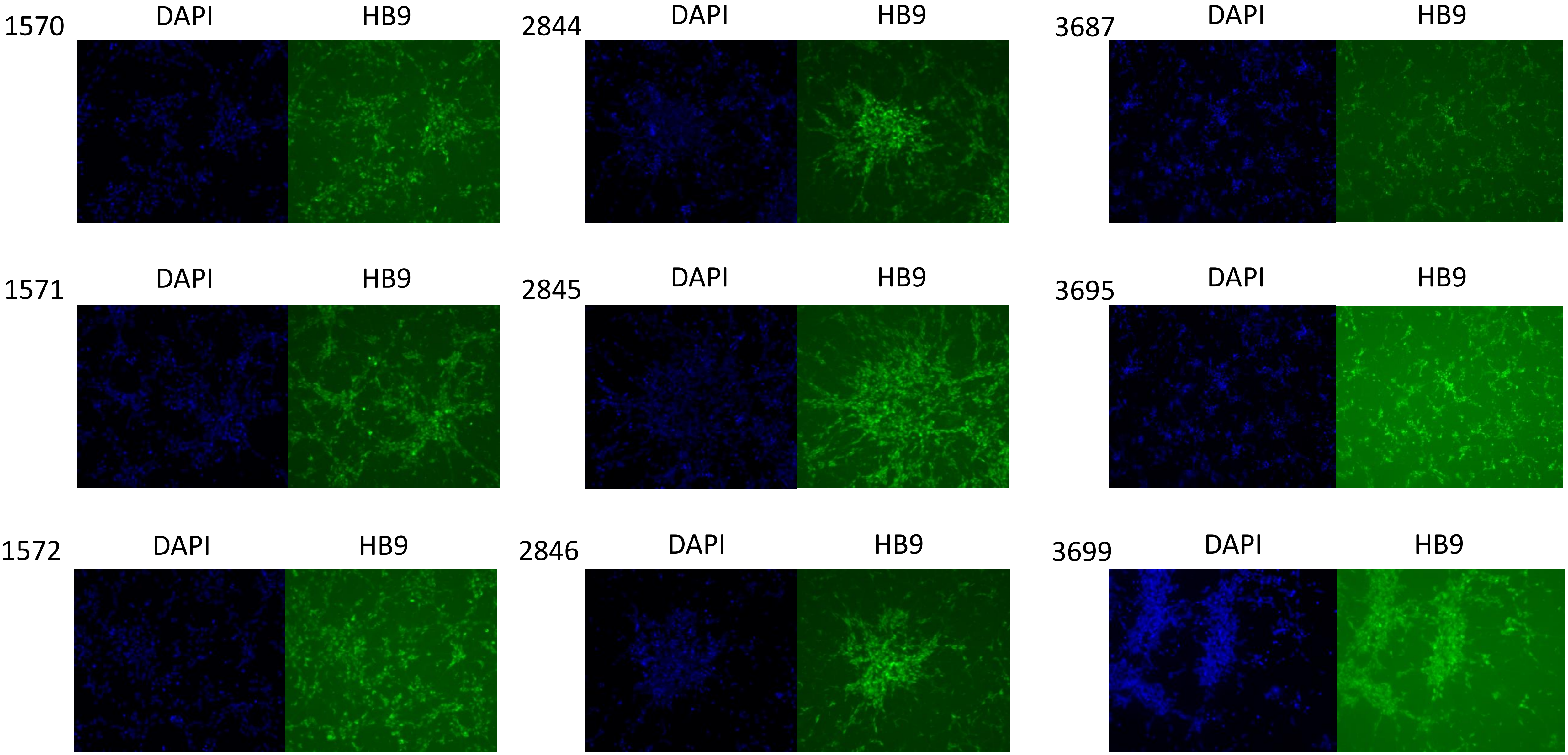

Fig. Immunofluorescent images of HB9 (green) and DAPI (blue) in $SOD1^{G85R}$ MNs.

Fig 1E. G85G

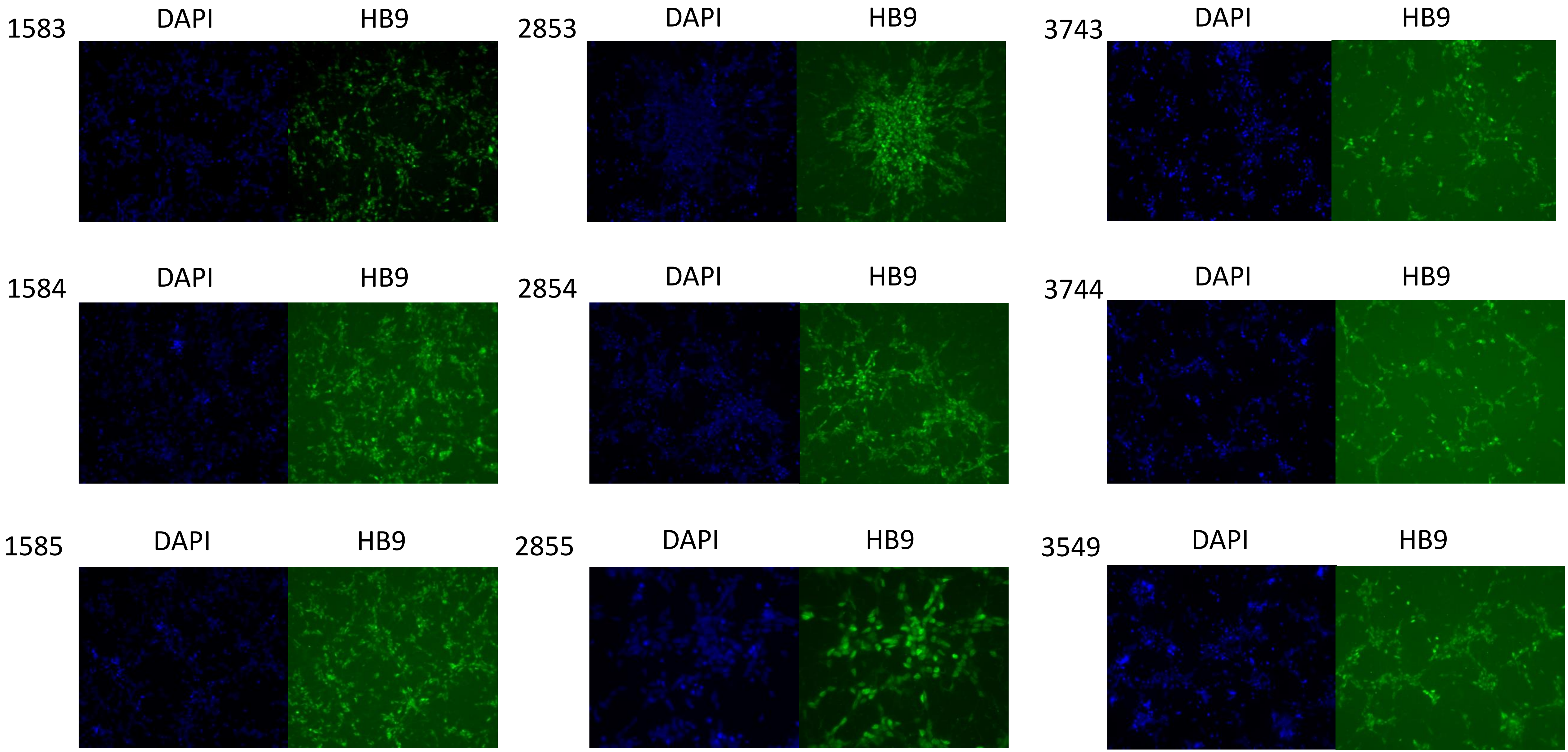

Fig. Immunofluorescent images of HB9 (green) and DAPI (blue) in SOD1^{G85G} MNs.

Fig 5A.

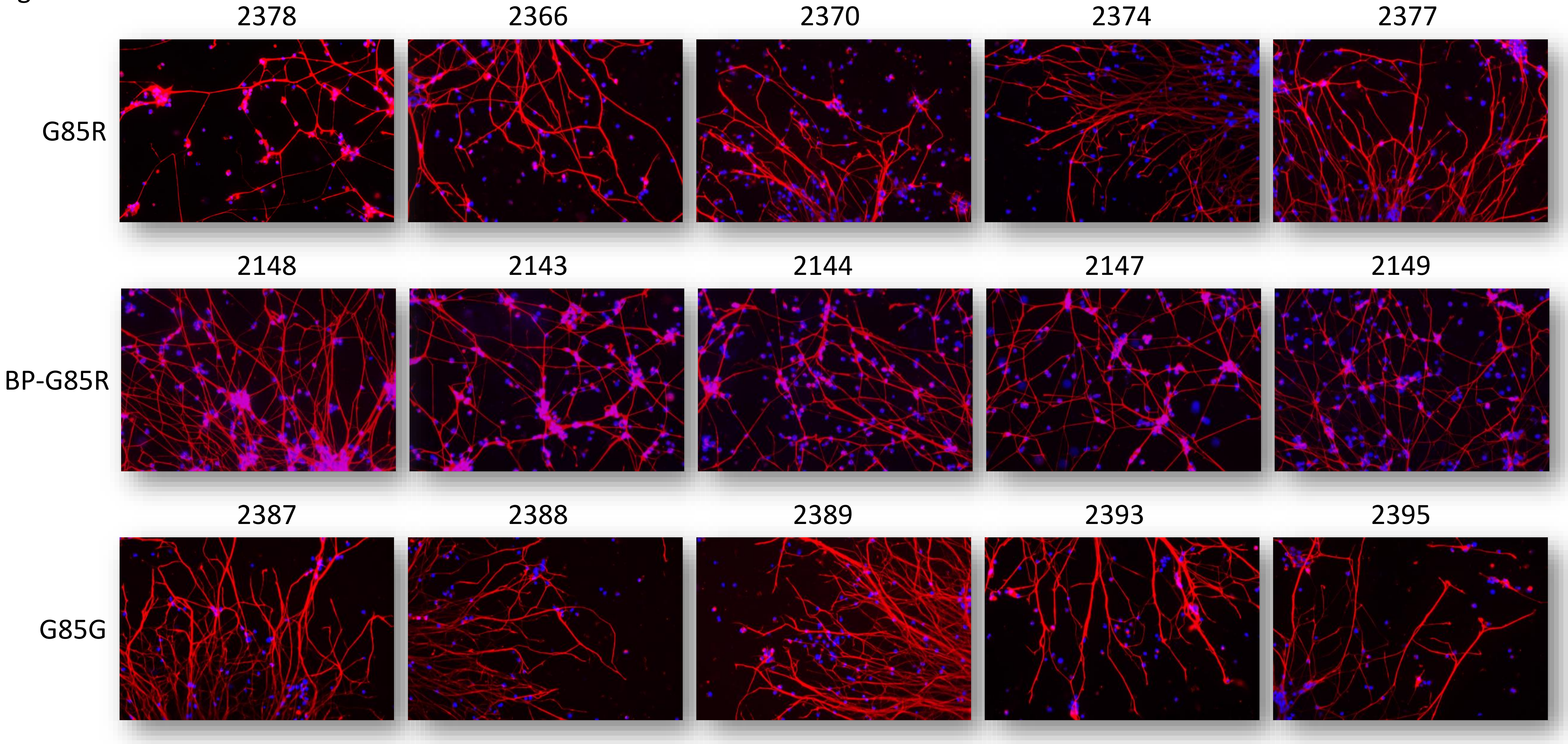

Fig. Immunofluorescent images of Neurofilaments (red) and DAPI (blue) in MNs.
