# Supplementary material for: n-Butylidenephthalide recovered calcium homeostasis to ameliorate neurodegeneration of motor neurons derived from amyotrophic lateral sclerosis iPSCs
Source: PLoS One. 2024 Nov 7;19(11):e0311573. doi: 10.1371/journal.pone.0311573 (PMC11542850; doi:10.1371/journal.pone.0311573)
Supplement: S1 Raw images — (PDF) [file pone.0311573.s004.pdf]

Fig. 3A NMDAR1 (105 KDa)

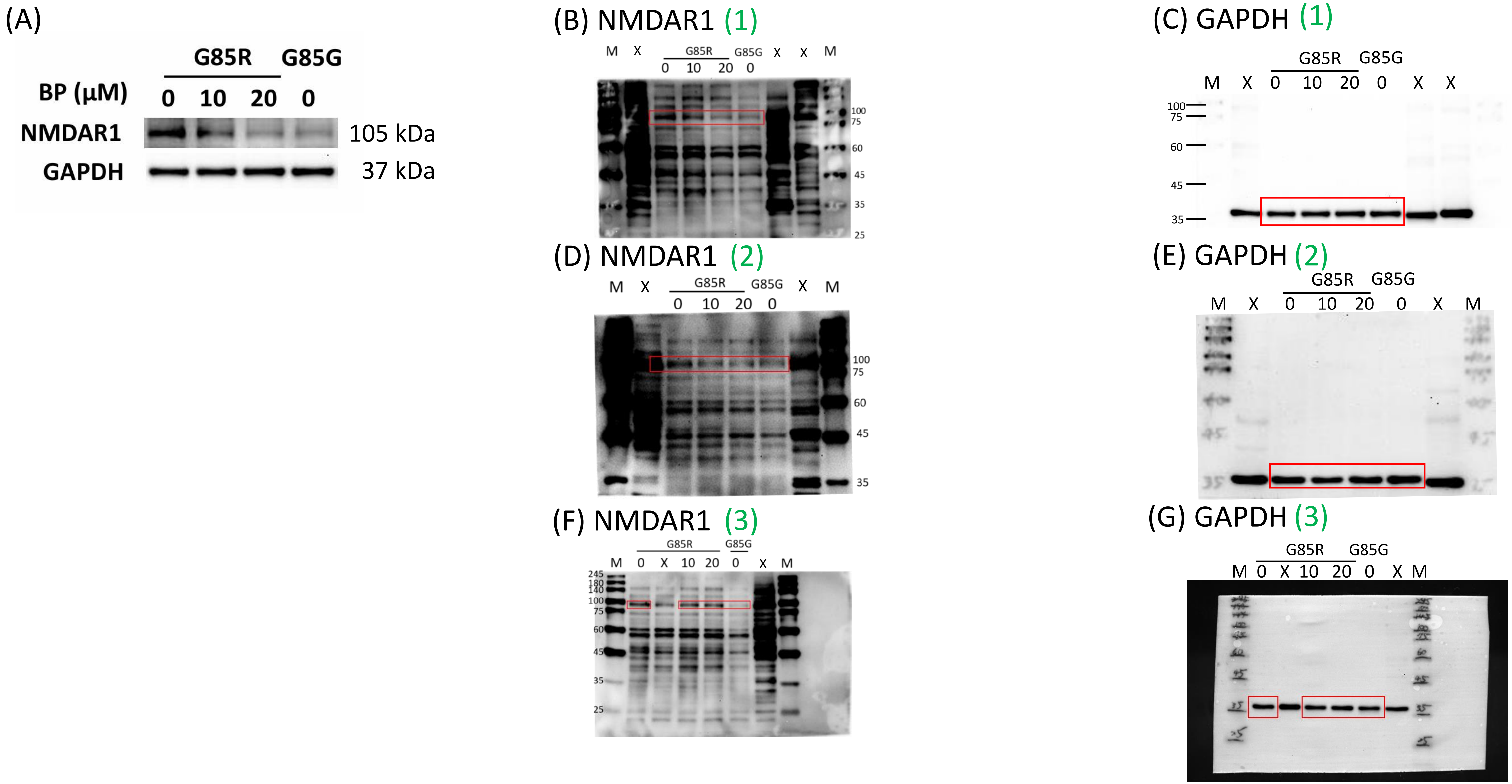

Fig. Western Blotting for NMDAR1 was performed in three replicates. (A) This figure showed the results presented in manuscript. The original imaging data of NMDAR1 (B, D and F) and GAPDH(C, E and G).

Fig. 3C GluR3 (100 KDa)

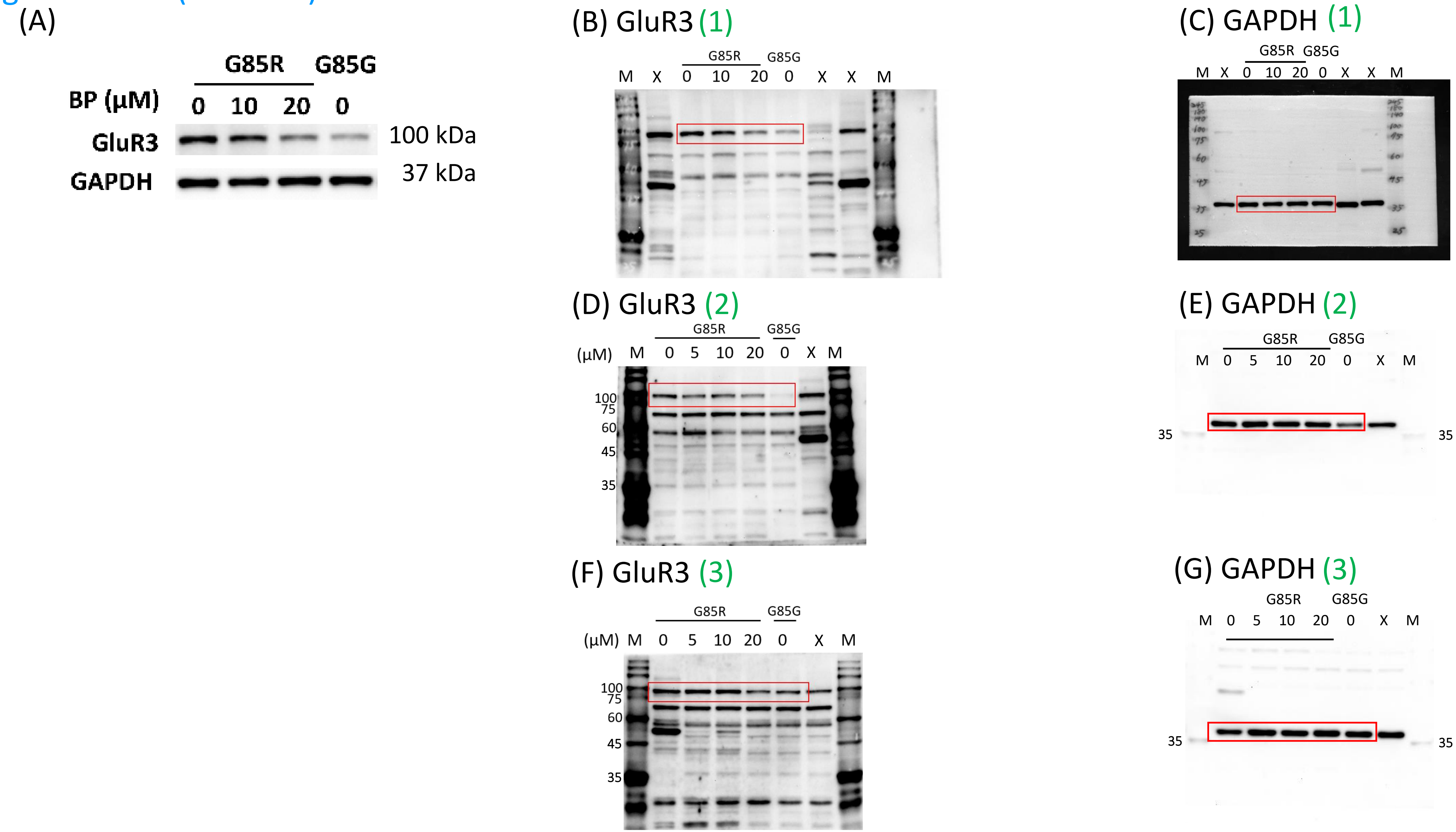

Fig. Western Blotting for GluR3 was performed in three replicates. (A) This figure showed the results presented in manuscript. The original imaging data of GluR3 (B, D and F) and GAPDH(C, E and G).

Fig. 5C activated caspase 3 (17 KDa)

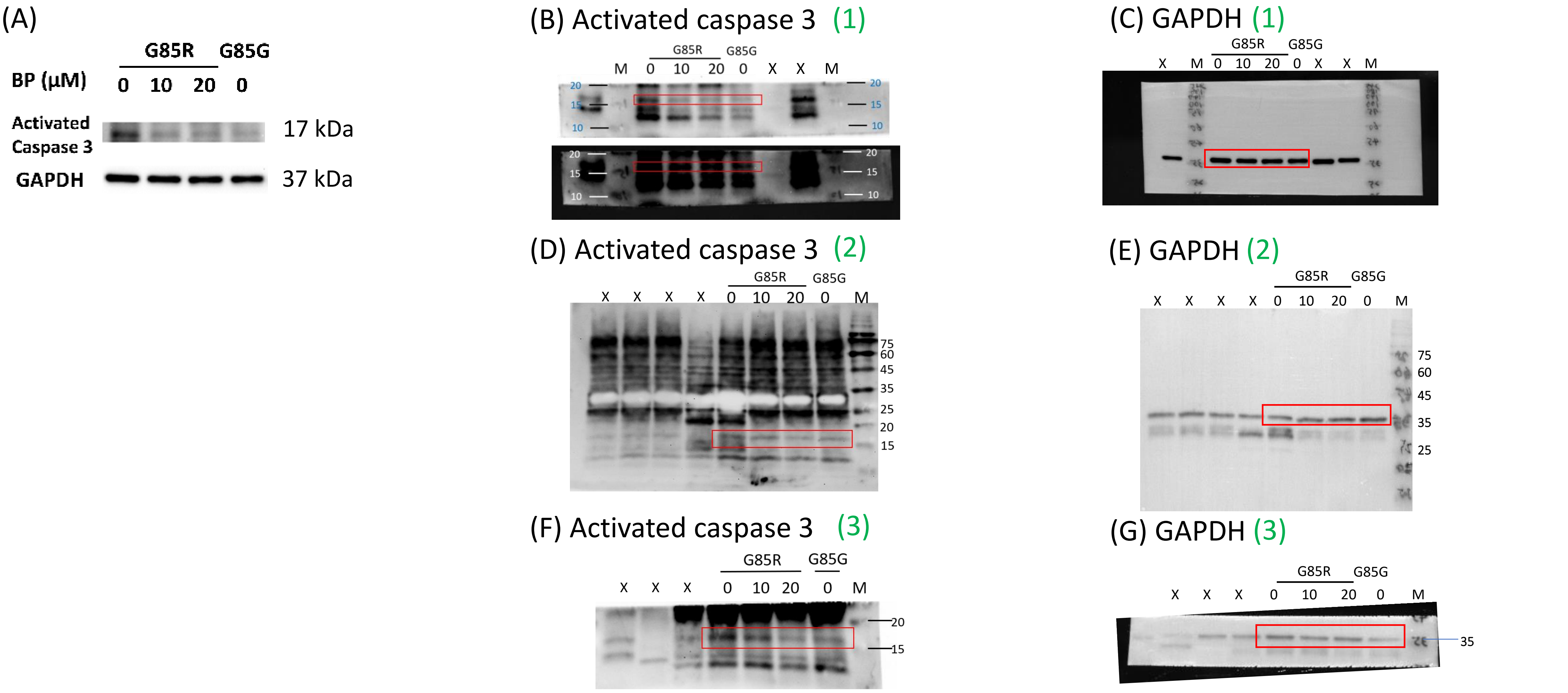

Fig. Western Blotting for activated caspase 3 was performed in three replicates. (A) This figure showed the results presented in manuscript. The original imaging data of NMDAR1 (B, D and F) and GAPDH(C, E and G). Due to the high signal of the protein in the upper half of the PVDF membrane, it may interfere with the visualization of the target protein. To mitigate this, we aim to preserve the presentation range as much as possible and focus on the lower membrane for visualization.

Fig. 6A LC3BII (13 KDa)

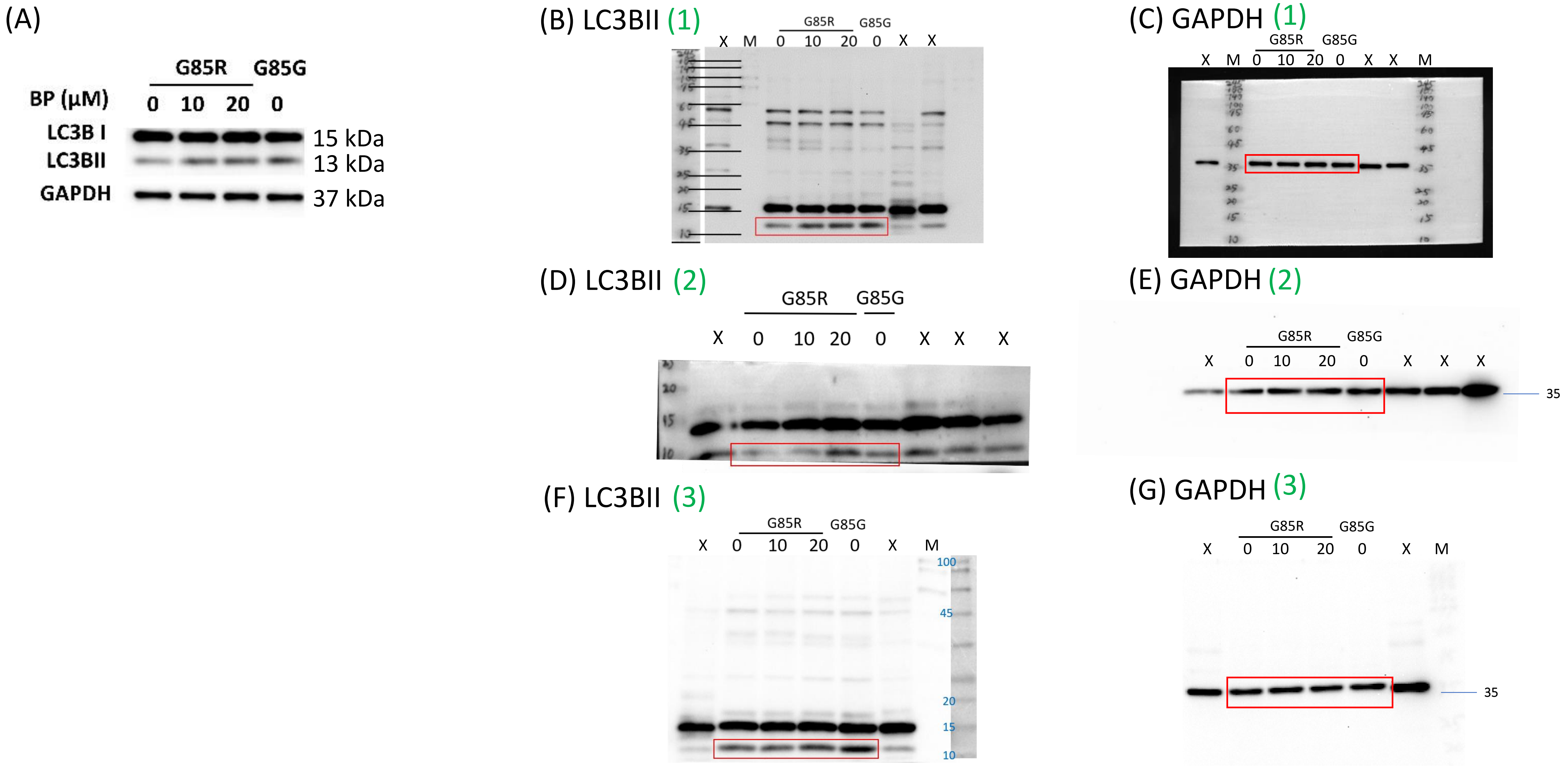

Fig. Western Blotting for LC3BII was performed in three replicates. (A) This figure showed the results presented in manuscript. The original imaging data of LC3BII (B, D and F) and GAPDH(C, E and G).

Fig. 6C p62 (62 KDa)

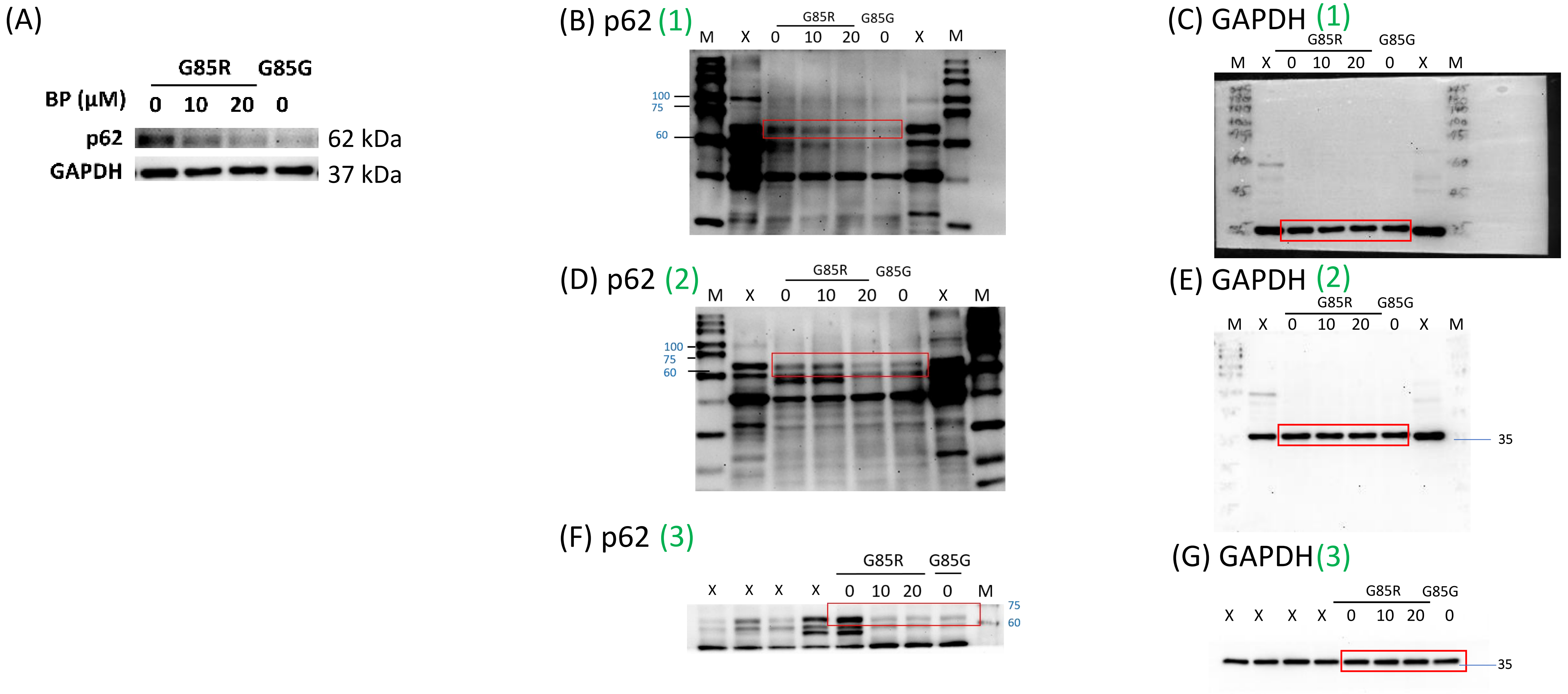

Fig. Western Blotting for p62 was performed in three replicates. (A) This figure showed the results presented in manuscript. The original imaging data of p62 (B, D and F) and GAPDH(C, E and G).
